# Comparison of Viremia, Cloacal Virus Shedding, Antibody Responses and Pathological Lesions in Adult Chickens, Quails, and Pigeons Infected with ALV-A

**DOI:** 10.1038/s41598-019-39980-y

**Published:** 2019-02-28

**Authors:** Zhongsheng Zhang, Weiguo Hu, Baoquan Li, Ru Chen, Weixing Shen, Huiling Guo, Huijun Guo, Hongmei Li

**Affiliations:** 1 Shandong Provincial Key Laboratory of Animal Biotechnology and Disease Control and Prevention, Taiàn, 271018 China; 2 College of Animal Science and Veterinary Medicine, Shandong Agricultural University, Taiàn, 271018 China; 3 Forest Farm of Mount Tai, Taiàn, 271018 China

## Abstract

Subgroup A of the avian leukosis virus (ALV-A) can cause severe pathological lesions and death in infected chickens, and its reported hosts have increased recently. To assess the susceptibility of adult chickens, quails, and pigeons to ALV-A, three sets of 250-day-old birds were intraperitoneally inoculated with ALV-A. Viremia and cloacal virus shedding were dynamically detected using an immunofluorescence assay (IFA), ALV-P27 antigen ELISA or RT-PCR; pathological lesions were assessed using tissue sections; ALV-A in tissues was detected by IFA; and ALV-A antibody responses were detected using antibody ELISA kits and an immune diffusion test. The results indicated that persistent viremia occurred in 80% (8/10) of infected chickens, and transient viremia occurred in 17% (2/12) of infected quails, but no viremia occurred in infected pigeons. Cloacal virus shedding occurred intermittently in 80% (8/10) of infected chickens and in 8% (1/12) of infected quails but did not occur in infected pigeons. Severe inflammatory pathological lesions occurred in the visceral tissues of most infected chickens, and mild lesions occurred in a few of the infected quails, but no pathological lesions occurred in the infected pigeons. The ALV-A virus was detected in the visceral tissues of most infected chickens but not in the infected quails and pigeons. Obviously different ALV-A antibody responses occurred in the infected chickens, quails and pigeons. It can be concluded that adult chickens, quails and pigeons have dramatically different susceptibilities to ALV-A. This is the first report on artificial infection by ALV-A in different birds.

## Introduction

Avian leukemia viruses (ALVs) are members of retrovirus family and have been classified into 11 subgroups^1–3^. Subgroup A of ALV (ALV-A) is an exogenous ALV that can cause viremia, immunosuppression, severe pathological lesions, tumorigenesis, and death in infected chickens^3,4^ and can cause great economic losses to the poultry industry^2,5^. There are currently no effective vaccines or drugs for controlling ALV-A infection.

ALV-A has been reported in both meat and layer chickens in the past few decades^6–9^. Recently, the virus was reported in some adult wild birds, such as the Eurasian wigeon, green-winged teal, and Baikal teal, which were found dead of unnatural causes in Northeast China^10–12^. The ALV P27 antigen has also been detected in the albumin of a small proportion of quail eggs (5/360) but not in the albumin of ducks’ (0/507) or geese’s (0/330) eggs (unpublished data). These results suggest that some birds other than chickens are likely to carry and spread ALVs, which may present great challenges for the prevention and control of ALVs and represent a serious threat to ecological stability. More attention should be paid to the spread and pathogenicity of ALV-A in different birds.

Like chickens, quails and pigeons are important domestic bird species that have been reared on a large scale worldwide. Little is known about their susceptibility to ALV-A strains isolated from chickens or their ability to transmit ALV-A strains. The results of many clinical cases showed that adult chickens, especially at peak egg laying, had high incidences of avian leukemia and could easily shed viral particles into their eggs through their reproductive ducts or cloacas^4–6^. To compare susceptibility to ALV-A among adult chickens, quails, and pigeons, 250-day-old quails, pigeons, and chickens were artificially infected with ALV-A. Then, viremia, cloacal virus shedding, pathological lesions and antibody responses were assessed at different days post infection. Some novel results were obtained.

## Materials and Methods

### Virus strain

The ALV-A-SDAU09C1 (GenBank: HM452339) strain was isolated from meat breeder chickens^9^ and provided by Professor Cui Zhizhong. The 50% tissue culture infective dose (TCID_50_) of ALV-A was determined using a limiting dilution assay in a 96-well plate covered with chicken embryo fibroblast (CEF) from 9-day-old chicken embryos, according to the Reed-Muench method. The positive cells were identified using an indirect immunofluorescence assay (IFA) mediated by a monoclonal antibody (MAb) specific for ALV-A^13,14^.

### Birds

Female pigeons were purchased from Tiancheng Pigeon Breeder Co. Ltd., Zibo, China. Female quails were purchased from Hebei Province Weiye Quail Breeder Co. Ltd. Hyline Brown layer hens were purchased from Dongyue Breeder Co. Ltd, Tai’an, China. All the birds were 250-days-old and housed in a clean and comfortable room. Before the start of the experiment, blood samples from the birds were collected and analyzed for the presence of ALV viruses and antibodies using ALV P27 ELISA test kits and ALV-antibody ELISA test kits (IDEXX USA Inc., Beijing, China), respectively. The birds that tested negative for ALV P27 antigen and ALV antibody were used in the study.

### Experimental design and ethics statement

A total of 18 quails, 16 pigeons, and 16 hens were randomly divided into the control group and ALV-A-infected group. Each bird in the ALV-A-infected group was inoculated intraperitoneally with 10^6^ TCID_50_ of the ALV-A-SDAU09C1 strain, and the control group was inoculated with PBS. Blood samples and cloacal swab samples from all the birds were collected every 3 days post inoculation (dpi) and tested for the ALV P27 antigen. At 21 dpi, all the birds were euthanized using the CO_2_ inhalation method, and the visceral organs of the birds, such as the liver, spleen and heart, were examined for pathological lesions.

The Animal Ethics Committee of the Shandong Animal Protection and Welfare Institute approved the experiments (Number: SDAU-2014-009), and all procedures related to the animals and their care conformed to the internationally accepted principles in the Guidelines for Keeping Experimental Animals issued by the government.

### ELISA and IFA for viremia

ALV viremia in the birds was detected using ELISA and IFA according to published methods^13,15^. Briefly, plasma samples from each bird were serially diluted and added to a monolayer of DF1 cells in a 24-well plate. Following incubation at 38.5 °C and 5% CO_2_ for 5 days, the supernatants of DF1 cells were tested for the presence of ALV by using IDEXX ALV P27 antigen ELISA kits. The relative antigen titer was determined by calculating the sample-to-positive (S/P) ratio using the formula [(mean of sample optical density) − (mean of negative control optical density)]/[(mean of positive control optical density) − (mean of negative control optical density)]. Each sample was tested in triplicate, and plasma samples with an S/P ratio ≥ 0.2 were considered virus-positive. All of the samples were further identified using IFA with an MAb specific for ALV-A in DF1 cells. The fluorescence signals in the positive IFA images were quantified using ImageJ software, and the number of cells was determined by the nuclear staining method. The viral titers or TCID_50_ values of ALV-A in plasma were calculated according to published methods^13^.

### ELISA and real-time RT-PCR fluorescence-based assays for cloacal virus shedding

To evaluate virus shedding from the cloacas of infected birds, ALV-A in the cloacal swab samples was detected using an IDEXX ALV P27 antigen ELISA kit^15^ and a real-time reverse transcription-polymerase chain reaction (RT-PCR) fluorescence-based assay, as previously described by Zhang *et al*.^16^. In brief, 1 ml of the dilution fluid from the kits was added to the cloacal swab samples, and three freeze-thaw cycles were performed. After sedimentation, the supernatants of the samples were evaluated for ALV P27 antigens in accordance with the manufacturer’s protocol. The positive ratios for ALV-A were calculated according to the positive standard of the kit.

In the fluorescence-based real-time RT-PCR assay, total RNA was directly extracted from the collected cloacal swab samples using a MicroElute Viral DNA/RNA Kit (Tiangen Biotech Co. Ltd., Beijing). The primers for ALV-A (forward: 5′-GGGAGGGGGAAATGTAGT-3′, reverse: 5′-GTTCGCAATCGTTAGGGA-3′) from the *env* gene of ALV-A-SDAU09C1 (GenBank: HM452339) were used for the specific detection of ALV-A proviral DNA, which generated a PCR product with a size of 262 bp. A probe (5′-CGTCATTCCTTCCTTATCTAGTCGCCAC-3′) with a FAM fluorophore at the 5′ end and BHQ-1 at the 3′ end was used. The real-time RT-PCR conditions included an initial step at 42 °C for 5 min and 95 °C for 10 s and a second step of 40 cycles at 95 °C for 60 s and at 60 °C for 34 s. The fluorescent signal was analyzed, and the viral content of each sample was calculated according to the standard curve for ALV-A.

### Assessment of pathological lesions

Throughout the experiment, the birds were observed daily for behaviors and signs of illness. At 21 dpi, the infected birds were assessed for pathological lesions, including tumors and hyperplasia in the main visceral organs. Tissue sections of the livers, hearts and kidneys were prepared and stained with hematoxylin-eosin (H.E.) according to a protocol described elsewhere^17^, and these sections were observed and analyzed with a microscope. Microscopic lesions were determined based on previous scoring methods^18,19^. The scoring criteria for liver lesions were as follows: 0 = normal hepatic tissue; 1 = mild lesions (with congestion, lymphocyte infiltration, hepatocyte regeneration, etc.); 2 = severe inflammatory lesions (with steatosis, amyloidosis, necrosis, etc.); and 3 = typical hepatic lymphosarcoma.

### IFA for ALV-A in visceral tissues

ALV-A in the liver, spleen, and heart of each bird at 21 dpi was detected by IFA using a MAb specific for the ALV-A gp85 protein^20^. Briefly, frozen sections of the tissues were prepared and fixed with acetone at 4 °C for 15 min. After three washes with PBS, the sections were incubated with 10% fetal bovine serum for 1 h. After discarding the serum, the sections were added to the ALV-A MAb (diluted 1:20) directly without washing and incubated at 37 °C for 2 h. After being washed with PBS three times, the sections were incubated with goat anti-mouse antibodies labeled with FITC (diluted 1:200) in a wet box at 37 °C for 1 h. After washing with PBS three times, 50% PBS-glycerol was added to the sections for mounting. The fluorescent signals in the entire imaging area were analyzed using ImageJ software.

To further determine the TCID_50_ of ALV-A in these visceral tissues, the rest of the detected tissue samples were homogenized, filtered, and inoculated into DF1 cells. The TCID_50_ of ALV-A was determined using a limiting dilution assay according to the previously described method in the Virus Strain section.

### ELISA and immune diffusion testing for ALV-A serum antibodies

ALV-A antibodies in the collected serum samples from all of the birds were detected using an IDEXX ALV-A/B antibody test kit according to the published method^20^. The relative antibody titers in the serum were determined by calculating the S/P ratio [(mean of sample optical density) − (mean of negative control optical density)]/[(mean of positive control optical density) − (mean of negative control optical density)]. Each sample was tested in triplicate, and serum samples with an S/P ratio ≥ 0.4 were considered ALV-A/B antibody positive.

The combination of serum antibodies from the infected birds with the gp85 protein of ALV-A-SDAU09C1 was detected with an immune diffusion test^21^. Agar Petri dishes and sample wells were prepared, and the purified recombinant gp85 protein (rgp85) of ALV-A-SDAU09C1^20^ was placed in the center sample well. Standard positive serum and dilute serum samples were alternately placed in the surrounding sample wells. Agar Petri dishes were inverted in a humidified plate and freely diffused at 37 °C for 36 h. Serum samples with a precipitation line between the sample well and positive well were positive, and serum samples without precipitation were negative. The highest dilution of the serum sample with positive precipitation was defined as the antibody titer.

### Statistical analysis

All data are expressed as the mean ± standard deviation (SD). Intergroup differences were analyzed with ANOVA software, followed by Student-Newman–Keuls tests for multiple comparisons. P < 0.05 was considered to be statistically significant.

## Results

### Assessment of ALV-A viremia in adult chickens, quails and pigeons

**Figure 1:**
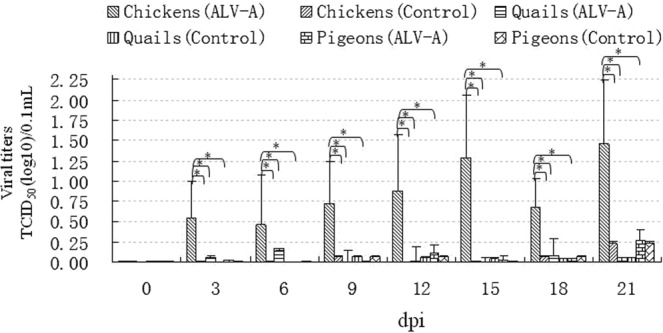
Viral titers of ALV-A in the plasma of chickens, quails and pigeons on different days post infection with ALV-A strain. dpi, days post infection; *P < 0.05 between two groups; Number of chickens is 10 (ALV-A group) and 6 (control group); Number of quails is 12 (ALV-A group) and 6 (control group); Number of pigeons is 10 (ALV-A group) and 6 (control group); the sample with TCID_50_/0.1 mL ≥ log10^0.63^ is positive to ALV-A.

To assess the viremia caused by ALV-A infection in each bird, serially diluted plasma samples were added to DF1 cells, and ALV-A titers in the supernatants were detected using an ALV P27 antigen ELISA kit at 5 days post inoculation. At the same time, ALV-A in infected DF1 cells was detected by IFA using an ALV-A MAb. The results of the ELISA in Fig. 1 show that the viral titers in ALV-A-infected chickens increased with increasing time post infection and were significantly higher than those in control chickens. However, the viral titers in ALV-A-infected quails and ALV-A-infected pigeons exhibited no dramatic differences compared with those in the control birds from 0 dpi to 21 dpi and were significantly lower than those in ALV-A-infected chickens.

**Figure 2:**
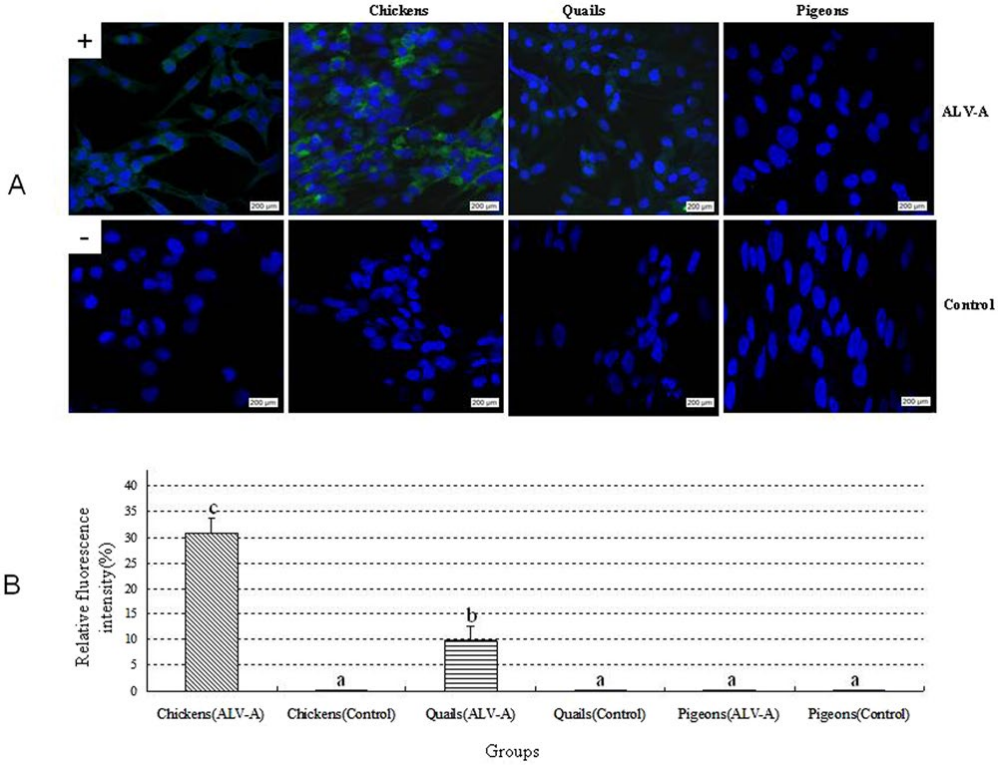
(**A**), IFA for detecting ALV-A in the plasma samples from different groups using DF1 cells and nuclear staining using DAPI (400x); (**B**), the relative fluorescence intensity in different groups was quantified using image J software. All the DF1 cells were detected using IFA mediated MAb against ALV-A on 5 day post inoculation. Green fluorescence in the cytoplasm: positive for ALV-A. +: positive control; −: negative control. In IFA of figure A, the plasma sample from ALV-A-infected chickens on 15 dpi was diluted to 1:100; all the plasma samples from control chickens on 15 dpi and from ALV-A-infected or control quails on 6 dpi and from ALV-A-infected or control pigeons on 15 dpi were not diluted. Different letters on the bars of two groups in figure B mean statistically significant difference between them (P < 0.05) and same letters mean no statistically significant difference (P > 0.05).

The results of the IFA in Fig. 2A show that the DF1 cells inoculated with the plasma samples from eight ALV-A-infected chickens and two ALV-A-infected quails had green fluorescence in the cytoplasm, and the results of analysis using Image J software in Fig. 2B show that the fluorescence intensity in the DF1 cells inoculated with ALV-A-infected chicken samples was dramatically higher than that in the DF1 cells inoculated with ALV-A-infected quail samples or pigeon samples. These findings suggest that the ALV-A content in plasma from ALV-A-infected chickens was much higher than that in plasma from ALV-A-infected quails. However, all plasma samples from ALV-A-infected pigeons were negative for ALV-A.

The ALV-A-positive ratios of the infected birds on different days post infection are presented in Table 1. The results show that the ALV-positive ratio in the infected chicken group was 40% (4/10) from 3 dpi to 9 dpi and 80% (8/10) from 15 dpi to 21 dpi. In the infected quail group, the ratio was 0% (0/12) from 3 dpi to 21 dpi, except for 6 dpi (17%, 2/12 birds). In the infected pigeon group, the ratio was 0% (0/10) from 0 dpi to 21 dpi. These results suggest that ALV-A infection induced persistent viremia in most adult chickens and transient viremia in a few of the adult quails, but no viremia was detected in adult pigeons.

**Table 1:** The positive ratios of viremia in chickens, quails, and pigeons on different days post infection with ALV-A.

Birds	Groups	0 dpi	3 dpi	6 dpi	9 dpi	12 dpi	15 dpi	18 dpi	21 dpi
Chickens	ALV-A	0% (0/10)	40% (4/10)	40% (4/10)	40% (4/10)	60% (6/10)	80% (8/10)	80% (8/10)	80% (8/10)
	Control	0% (0/6)	0% (0/6)	0% (0/6)	0% (0/6)	0% (0/6)	0% (0/6)	0% (0/6)	0% (0/6)
Quails	ALV-A	0% (0/12)	0% (0/12)	17% (2/12)	0% (0/12)	0% (0/12)	0% (0/12)	0% (0/12)	0% (0/12)
	Control	0% (0/6)	0% (0/6)	0% (0/6)	0% (0/6)	0% (0/6)	0% (0/6)	0% (0/6)	0% (0/6)
Pigeons	ALV-A	0% (0/10)	0% (0/10)	0% (0/10)	0% (0/10)	0% (0/10)	0% (0/10)	0% (0/10)	0% (0/10)
	Control	0% (0/6)	0% (0/6)	0% (0/6)	0% (0/6)	0% (0/6)	0% (0/6)	0% (0/6)	0% (0/6)

(Plasma samples with green fluorescence detected in the cytoplasm of DF1 cells by IFA were positive for ALV-A).

### Assessment of cloacal ALV-A shedding in adult chickens, quails and pigeons

The shedding of ALV in the cloacas of infected birds was detected using an ALV P27 antigen ELISA. The positive ratios for cloacal virus shedding in the three types of birds on different days post infection with ALV-A were calculated and listed in Table 2. The results show that the positive ratios in ALV-A-infected chickens were 80% (8/10) at 15 dpi and not more than 40% on other days post infection. Among the ALV-A-infected quails, only one bird was positive at 3 dpi and 12 dpi. These results suggest that the cloacal virus shedding in these infected birds was intermittent and not persistent.

**Table 2:** The positive ratios of cloacal virus shedding in chickens, quails, and pigeons on different days post infection with ALV-A.

Birds	Groups	0 dpi	3 dpi	6 dpi	9 dpi	12 dpi	15 dpi	18 dpi	21 dpi
Chickens	ALV-A	0% (0/10)	0% (0/10)	20% (2/10)	40% (4/10)	20% (20/10)	80% (8/10)	20% (2/10)	20% (2/10)
	Control	0% (0/6)	0% (0/6)	0% (0/6)	0% (0/6)	0% (0/6)	0% (0/6)	0% (0/6)	0% (0/6)
Quails	ALV-A	0% (0/12)	8% (1/12)	0% (0/12)	0% (0/12)	8% (1/12)	0% (0/12)	0% (0/12)	0% (0/12)
	Control	0% (0/6)	0% (0/6)	0% (0/6)	0% (0/6)	0% (0/6)	0% (0/6)	0% (0/6)	0% (0/6)
Pigeons	ALV-A	0% (0/10)	0% (0/10)	0% (0/10)	0% (0/10)	0% (0/10)	0% (0/10)	0% (0/10)	0% (0/10)
	Control	0% (0/6)	0% (0/6)	0% (0/6)	0% (0/6)	0% (0/6)	0% (0/6)	0% (0/6)	0% (0/6)

(Samples with S/P ≥ 0.2 were positive according to the criteria of the ALV P27 antigen IDEXX ELISA test kit).

**Figure 3:**
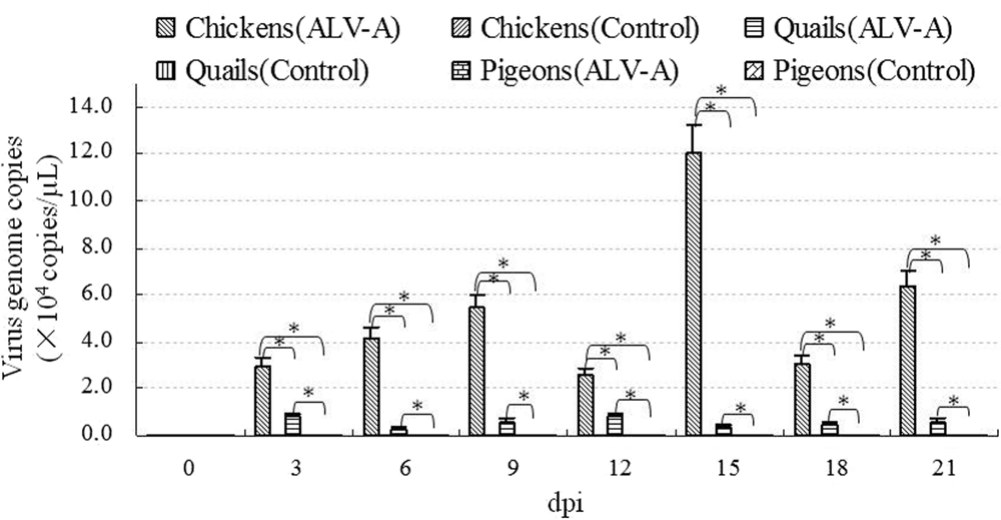
ALV-A copies in the cloacal swabs from the chickens, quails and pigeons on different days post infection with ALV-A strain. *P < 0.05 between two groups; Number of chickens is 10 (ALV-A group) and 6 (control group); Number of quails is 12 (ALV-A group) and 6 (control group); Number of pigeons is 10 (ALV-A group) and 6 (control group); each sample was detected using real-time fluorescent RT-PCR assay.

To further analyze the differences in cloacal virus shedding, the ALV-A content in all the cloacal swab samples was detected using a fluorescence-based real-time RT-PCR assay. The results in Fig. 3 show that the ALV-A content in the samples from infected chickens was significantly higher than that in the samples from infected quails and infected pigeons from 3 dpi to 21 dpi; moreover, the ALV-A content in the samples from infected quails was significantly higher than that in the samples from infected pigeons. These results suggested that ALV-A cloacal shedding clearly differed among the infected adult chickens, quails and pigeons.

### ALV-A infection caused different pathological lesions in adult chickens, quails and pigeons

**Figure 4:**
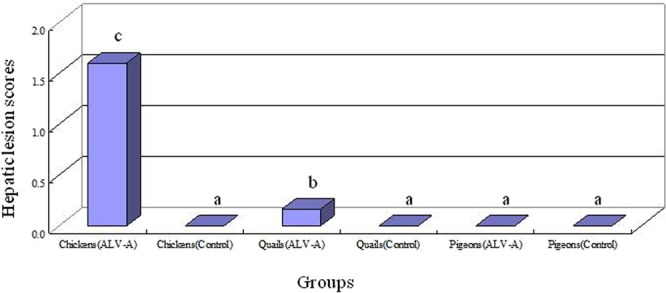
The hepatic lesion scores of different groups. The scoring criteria: 0 = normal hepatic tissue; 1 = mild lesions (including congestion, lymphocyte infiltration, hepatocyte regeneration); 2 = severe inflammatory lesions (including steatosis, amyloidosis, necrosis); and 3 = hepatic lymphosarcoma; data are presented as means ± SD; different letters on the bars of two groups mean statistically significant difference between them (P < 0.05) and same letters mean no significant difference (P > 0.05).

During the entire experimental timecourse up to 21 dpi, none of the infected birds showed any clinical signs of disease, tumors or death. Microscopically, no typical tumor cells were observed in sections of liver, heart or kidney from infected birds, but some inflammatory pathological lesions occurred in most of the infected chickens and in a few of the infected quails. The hepatic tissue sections from most of the infected chickens showed severe inflammatory pathological lesions, such as steatosis, cellular fusion, degeneration, and nuclear condensation; most of the infected quails did not show hepatic tissue pathological lesions, except for mild pathological lesions such as congestion, cytoplasmic degeneration, and nuclear hyperchromasia in a few of the infected quails. However, no pathological lesions were observed in any of the infected pigeons. The hepatic lesion scores of all the birds were calculated, and the results of the statistical analysis are presented in Fig. 4. The results showed that the lesion scores in ALV-A-infected chickens were 1.5~2.0, and were 0.0~0.5 in ALV-A-infected quails, and were almost zero in ALV-A-infected pigeons. These results suggested that ALV-A can caused different levels of pathological lesions in adult chickens, quails and pigeons, and caused more severe lesion in chickens than in quails or in pigeons.

### ALV-A was found in the visceral tissues of most infected chickens but not in infected quails and pigeons

**Figure 5:**
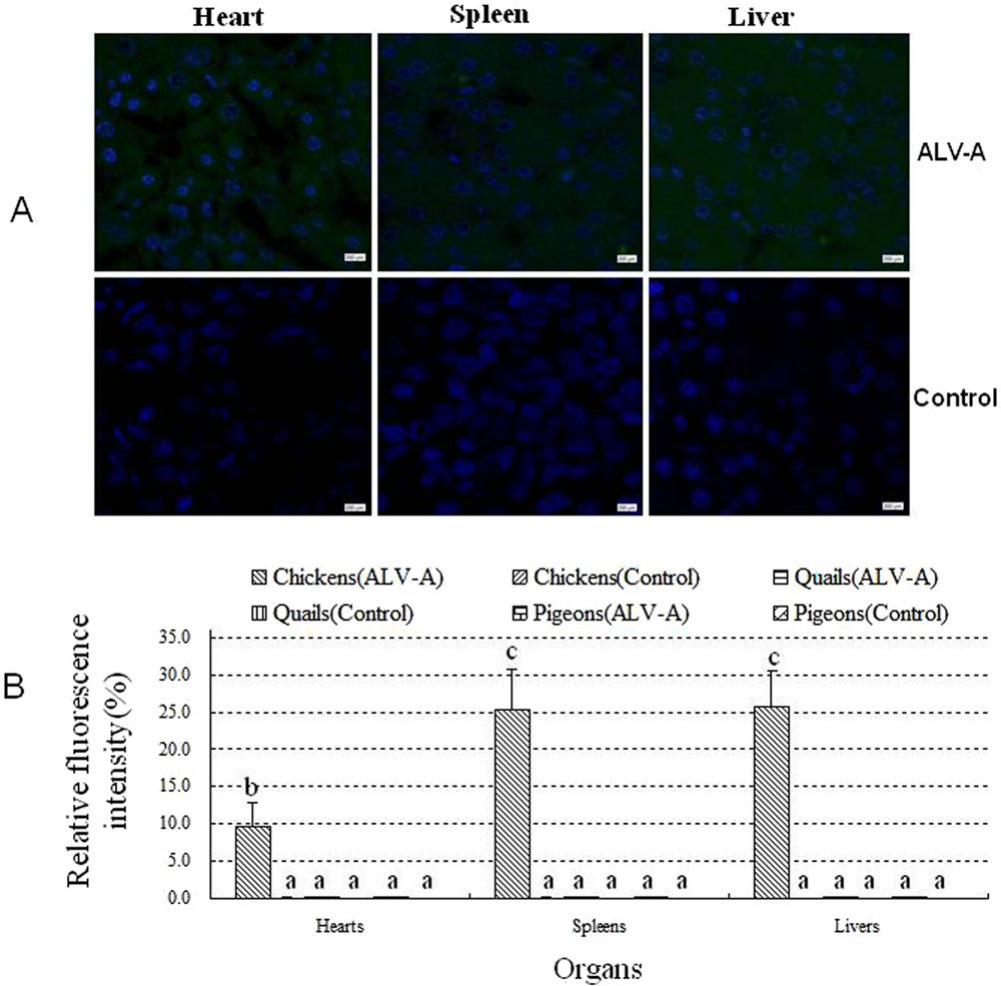
(**A**), IFA for ALV-A in the tissue sections of heart, spleen and liver from ALV-A-infected chickens on 21 dpi and nuclear staining using DAPI (400x); (**B)**, the relative fluorescence intensity in these organs was quantified using image J software. In figure A, green fluorescence in the cells showed positive for ALV-A in the corresponding organs. In figure B, Different letters on the bars of two groups mean statistically significant difference between them (P < 0.05) and same letters mean no statistically significant difference (P > 0.05).

To further confirm the existence of the ALV-A virus in the visceral tissues of infected birds, an MAb specific for ALV-A gp85 was used to detect viral antigen in the heart, spleen and liver on 21 dpi by IFA. The frozen sections of the hearts, spleens, and livers from a few of the infected chickens showed specific green fluorescence in the cytoplasm (Fig. 5A), which suggests that these tissues were positive for ALV-A. However, the sections of the organs from the infected quails and pigeons were not ALV-A positive. The fluorescence intensity of each section was quantified using Image J software, and all fluorescence intensities were statistically analyzed, as shown in Fig. 5B. The results show that the fluorescence intensities in the tissue sections of hearts, spleens and livers from infected chickens were significantly higher than those in the tissue sections of hearts, spleens and livers, respectively, from infected quails or pigeons.

The positive ratios and TCID_50_ of ALV-A in these examined tissue sections were calculated and are shown in Table 3. The results show that the positive ratios in the hearts, spleens and livers of the infected chickens were 40% (4/10), 80% (8/10) and 80% (8/10), respectively, and the TCID_50_ of ALV-A per gram in these positive tissues reached 10^3.2 ± 2.5^, 10^4.0 ± 3.4^, and 10^4.2 ± 3.6^, respectively. However, ALV-A tests were negative in all examined tissues from the infected quails and pigeons.

**Table 3:** The positive ratios of ALV-A in the hearts, spleens and livers of all the chickens, quails, and pigeons at 21 days post infection with ALV-A.

Birds	Groups	Hearts	Spleens	Livers
Chickens	ALV-A	40% (4/10)	80% (8/10)	80% (8/10)
	Control	0% (0/6)	0% (0/6)	0% (0/6)
Quails	ALV-A	0% (0/12)	0% (0/12)	0% (0/12)
	Control	0% (0/6)	0% (0/6)	0% (0/6)
Pigeons	ALV-A	0% (0/10)	0% (0/10)	0% (0/10)
	Control	0% (0/6)	0% (0/6)	0% (0/6)

(Organ samples with green fluorescence detected in the cytoplasm of frozen tissue sections were considered positive for ALV-A by IFA).

### ALV-A induced antibody responses in chickens and quails but not in pigeons

**Figure 6:**
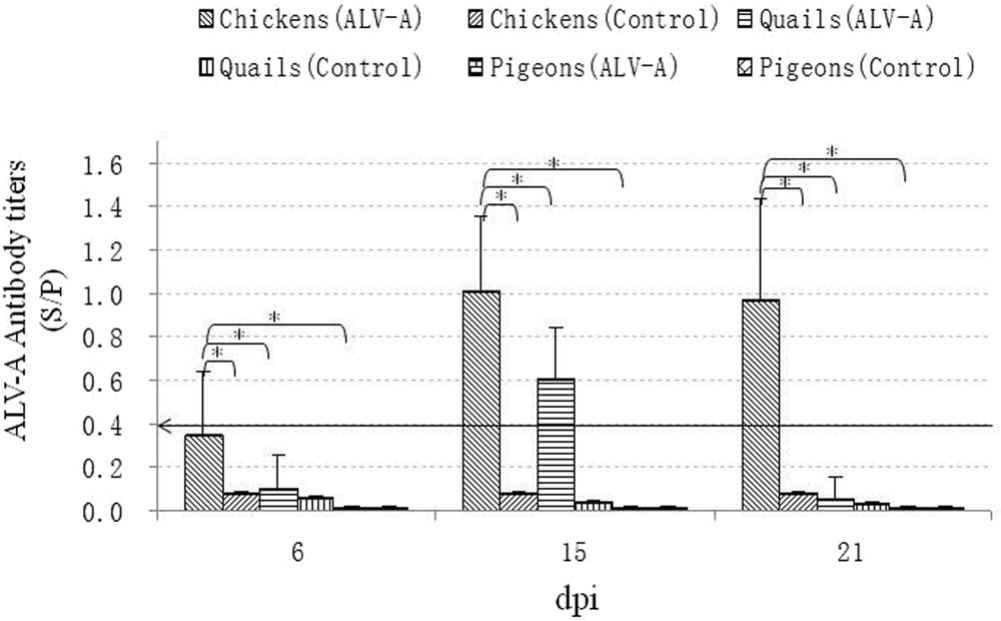
Antibody titers of ALV-A in the sera of the chickens, quails and pigeons on different days post infection with ALV-A strain. *P < 0.05 between two groups; Number of chickens is 10 (ALV-A group) and 6 (control group); Number of quails is 12 (ALV-A group) and 6 (control group); Number of pigeons is 10 (ALV-A group) and 6 (control group); the line with arrow means the cut off of antibody titers (S/P = 0.4).

ALV-A antibodies in the serum samples were detected using IDEXX ALV-A/B antibody ELISA kits on 6 dpi, 15 dpi, and 21 dpi. The results in Fig. 6 show that ALV-A antibodies could be detected in most of the infected chickens at 15 dpi and 21 dpi, and the positive ratios were up to 60% (6/10) according to the positive critical value of the ELISA kit (S/P > 0.4). The ALV-A antibody titers in the infected quails were dramatically lower than those in the infected chickens, and the positive ratios were up to 33% (4/12). However, ALV-A antibodies in the infected pigeons could not be detected because the antibody titers were below the detection limits of ELISA. The results of the immune diffusion test showed that the positive antisera from the infected chickens and quails could combine with the rgp85 protein antigen, and the diluted serum antibody titers reached 1:2^5^ and 1:2^3^, respectively. However, the antisera from the infected pigeons were not positive. These results suggested that the antibody responses to ALV-A infection clearly differed among adult chickens, quails and pigeons.

## Discussion

ALV-A is a tumorigenic RNA virus to which chickens are highly susceptible. ALV-A can cause growth retardation, immunosuppression, viremia, lymphocyte tumors, and even death in infected chickens and is one of the more dangerous pathogens in the chicken industry^2,3,22^. In recent years, ALV-A has also been detected in quails and wild birds in China^10,12^, but little is known about its pathogenicity in these birds or the susceptibility of these birds to ALV-A. In this study, the pathogenicity of ALV-A in adult chickens, quails and pigeons and the susceptibility of the birds to ALV-A were assessed.

ALV-A-SDAU09C1, which was isolated from meat breeder chickens by Dr. Zhang in 2009^9^, was intraperitoneally inoculated into adult chickens, quails, and pigeons. The pathogenicity of this viral strain in young chickens (embryonic or <7-day-old chickens) was systematically studied by Dr. Zhang Qing-chan in her PhD dissertation, and the virus was found to cause growth retardation, immunosuppression, and persistent viremia in most infected chickens and lymphoma in a few infected chickens from 22~27 weeks post infection (unpublished data). The pathogenicity of ALV-A in adult birds has not been reported until now. To improve the chances of infecting adult birds in this study, the dose of ALV-A was increased to 10^6^ TCID_50_ per bird, which is much higher than that used in young chickens in some previous studies^3,9^.

In this study, DF1 cells, a cell line from embryo fibroblast cells of C/E type chickens, in which exogenous ALVs such as ALV-A can replicate and grow but endogenous ALVs cannot^9,23^, were used to isolate and replicate ALV-A from the plasma samples. ALV P27 antigen ELISA and IFA using a MAb specific for ALV-A were used to detect ALV-A in the supernatant and cytoplasm of DF1 cells, respectively. These methods can not only specifically determine ALV-A in samples and eliminate the interference of other subgroups of ALV during the detection of ALV-A, but also effectively determine the biological concentration of ALV-A, such as its TCID_50_. The detection results showed that persistent viremia occurred in 80% (8/10) of infected chickens, and the viral titers were significantly higher in infected chickens than in control chickens (Figs 1, 2 and Table 1), which is consistent with the results obtained by Dr. Zhang in young chickens (unpublished data); in contrast, “transient” viremia occurred in 17% (2/12) of the infected quails, and their viral titers were much lower than those in the infected chickens. No viremia occurred in ALV-A-infected pigeons. These results suggest that ALV-A can cause more severe viremia in adult chickens than in adult quails or pigeons.

The reproductive duct and cloaca are important ALV-shedding channels in virus-positive chickens, and this can cause their offspring or other susceptible chickens to become infected^5^. Cloacal swabs and egg albumin have been recommended for screening virus-positive chickens in clinics^15,24^. In this study, ALV-A in cloacal swabs was used to determine viral shedding in three species of adult birds. The results showed that ALV-A could be intermittently detected in the cloacas of most infected chickens (8/10) and a few infected quails (1/12), but the viral titers were much lower in the infected quails than in the infected chickens. In the cloacas of all infected pigeons, ALV-A testing was negative. These results suggest that adult chickens can shed ALV-A particles from their cloacas more readily than adult quails, but adult pigeons cannot. This difference could explain why it is very difficult to detect ALV in quails or why the positive ratio was very low in our epidemiological investigation in quails (the positive ratio in quails’ eggs was 1.4% (5/360)).

In this study, the pathological lesions caused by ALV-A infection in the visceral tissues of infected birds were comparatively analyzed. Some previous studies indicated that pathological lesions, such as lymphoma, inflammatory lesions and congestion, caused by ALV-A mainly occurred in the livers, kidneys, spleens, and hearts; in particular, many typical lesions could be observed or detected in the liver^25–27^. However, there have been no reports regarding the pathological lesion scores of tumorigenic viruses until now. Here, the pathological lesions caused by tumorigenic viruses in the livers were classified into four grades from 0 to 3 according to previous studies and published methods for determining lesion scores during viral infection^18,19^. This method could easily quantify the pathological lesions in ALV-A-infected birds, and could objectively show different levels in pathological lesions among three sets of infected birds: the pathological lesions in the livers of ALV-A-infected chickens at 21 dpi ranged from grade 1 to grade 2 (scores = 1.5~2.0), and the lesions in ALV-A-infected quails ranged from grade 0 to grade 1 (scores = 0.0~0.5) and the lesions were almost grade 0 in all ALV-A-infected pigeons (scores = 0). In other words, although lymphoma or lymphoma cells did not occur in all infected adult birds, severe inflammatory lesions occurred in most infected chickens. In contrast, mild pathological lesions occurred in a few infected quails. However, no pathological lesions occurred in any of the infected pigeons. This can supply a new and scientific method for further study the pathological lesions caused by tumorigenic viruses.

ALV-A in the livers, spleens and hearts of the infected birds was detected using IFA. The results showed that a large amount of ALV-A occurred in the three tissues of the infected chickens but rarely occurred in the infected quails or pigeons (Fig. 5). These results indicated that ALV-A might have higher tropism to chicken tissues than quail or pigeon tissues. Additionally, the fluorescence intensities of IFA and the viral titers in these three tissues of infected chickens showed that the number of viral particles of ALV-A was much higher in the liver and spleen than in the heart. We deduced that ALV-A tends to accumulate or replicate more in the liver and spleen and causes serious pathological lesions in these tissues. In some clinical cases, typical tumors caused by ALV-A occur in the livers and spleens of chickens^10,28^. This observation may be related to the accumulation of a large amount of ALV-A in these tissues.

The antibody responses to ALV infection in chickens are very complex. Some studies confirmed that antibody responses induced by ALV infection occur in some chickens, but not in others due to immune tolerance^29,30^. In this study, an ALV-A antibody ELISA was used to detect serum antibodies, and an immune diffusion test was used to analyze the neutralization of serum antibodies. The results showed that immunoprecipitating antibodies against gp85 in the serum could be induced in most of the ALV-A-infected chickens and in some of the ALV-A-infected quails, but the antibody positive ratios and the antibody titers were significantly different between the two species of birds. However, in the infected pigeons, serum antibodies could not be detected. These results suggest that antibody responses caused by ALV-A infection are significantly different among the three species of adult birds.

There are few previous reports on the susceptibility of adult quails and pigeons to ALV-A virus isolated from chickens. Shen *et al*. reported that the ALV-J strain isolated from chickens could infect some quails and pheasants (the positive ratios were 10% (3/30) and 37.5% (6/16), respectively) after viral gene mutation through many generations of cross-species transmission from chickens to turkeys^31^. The results in this study suggested that adult quails were less susceptible to ALV-A than adult chickens and barely shed the virus, although a few of the infected birds showed “transient” viremia or cloacal virus shedding and mild lesions.

In the past century, it was reported that pigeon cells were resistant to most avian tumor viruses except ALV-D^32^, but there have been no experiments to confirm that pigeons are resistant to ALV-A infection until now. In this study, the results showed that none of the infected pigeons had viremia, cloacal virus shedding, antibody responses, typical clinical signs or other obvious pathological lesions, and ALV-A could not be detected in the visceral tissues or cloacal swabs of the pigeons. These results suggest that pigeons are more resistant to ALV-A than chickens and quails and cannot shed the virus from their cloacas.

No further experiments were performed to clarify the differences in resistance to ALV-A among chickens, quails, and pigeons in this study. We deduced that the genetic differences among species might cause the observed differences in their resistance to ALV. Some studies confirmed that quails possess the receptor TVA, which can recognize ALV-A^33,34^; however, this receptor is 65% homologous to that of chickens and has a lower affinity for ALV-A^35^. A charged second-site mutation in some amino acids of quail TVA can increase their affinity and susceptibility to ALV-A^36,37^. Differences in ALV receptor affinity could partly explain the difference in susceptibility to ALV-A between quails and chickens. There are no reports on pigeon TVA to date, and further studies are needed to determine the mechanism of pigeon resistance to ALV-A.

From this study, it can be concluded that ALV-A infection causes different levels of viremia, cloacal virus shedding, antibody responses and pathological lesions in adult chickens, quails, and pigeons. This finding suggests that there are differences in the susceptibility or resistance of these three bird species to ALV-A. This study is very helpful for understanding the pathogenicity and transmission of ALV-A among these different bird species.
